# Dedifferentiated liposarcoma of the gallbladder: first reported case

**DOI:** 10.1186/s12957-018-1520-5

**Published:** 2018-11-12

**Authors:** Adriano Carneiro da Costa, Fernando Santa-Cruz, Brena F. Sena, Ademar Lopes, Nicole Leite, Alexandre Rolim da Paz, Álvaro A. B. Ferraz, José-Luiz Figueiredo

**Affiliations:** 1 Oncological Surgery Unit, Napoleão Laureano Hospital, Av. Flamboyant, 198, Anatólia, João Pessoa, PB 58052-010 Brazil; 2 School of Medicine, Federal University of Pernambuco, Recife, PE Brazil; 3 Harvard T.H. Chan School of Public Health, Boston, MA USA; 4 Department of Sarcoma, A.C. Camargo Cancer Center, São Paulo, SP Brazil; 5 School of Medicine, Faculty of Medical Sciences of Paraíba, João Pessoa, PB Brazil; 6 Department of Pathology, Napoleão Laureano Hospital, João Pessoa, PB Brazil; 7 Department of Surgery, Federal University of Pernambuco, Recife, PE Brazil

**Keywords:** Gallbladder disease, Gallbladder neoplasms, Immunohistochemistry, Liposarcoma, Dedifferentiated liposarcoma

## Abstract

**Background:**

Liposarcoma of the gallbladder is an extremely rare sarcoma, with only five cases reported in the literature according to our knowledge.

**Case presentation:**

A 71-year-old woman was referred to the Surgical Oncology Division of Napoleão Laureano Hospital (João Pessoa, PB, Brazil) due to a solid mass at the right side of the abdomen and fever, with no signs of jaundice. Abdominal ultrasonography and computed tomography (CT) evidenced an extensive gallbladder lobular formation adhered to the inferior border of the right hepatic lobe and cholelithiasis. The CT report suggested gallbladder liposarcoma. A cholecystectomy associated with resection of segments IV-B and V of the liver were performed. Intraoperative frozen sections were compatible with gallbladder sarcoma. Anatomopathological examination and immunohistochemistry confirmed dedifferentiated liposarcoma with foci of heterologous leiomyosarcomatous differentiation and undifferentiated fusocellular areas of high histological grade.

**Conclusion:**

This is the first case of dedifferentiated liposarcoma of the gallbladder to be reported.

## Background

The gallbladder is the primary site of several types of malignant mesenchymal tumors, though all of these neoplasms are extremely rare [1, 2]. Patients usually present with pain in the right upper quadrant of the abdomen with or without jaundice, and generally, poor outcomes are expected [2, 3].

Liposarcoma is the second most common soft tissue sarcoma among adults, and their more frequent sites are the proximal extremities and the retroperitoneum [3–5]. Primary intra-abdominal liposarcomas are rare, and they are more commonly located in the mesentery and peritoneum [4]. These tumors tend to invade nearby structures and to spread within the abdominal cavity [1].

Here, we report a case of dedifferentiated liposarcoma, the first report of this histological subtype in the gallbladder.

## Case presentation

**Fig. 1:**
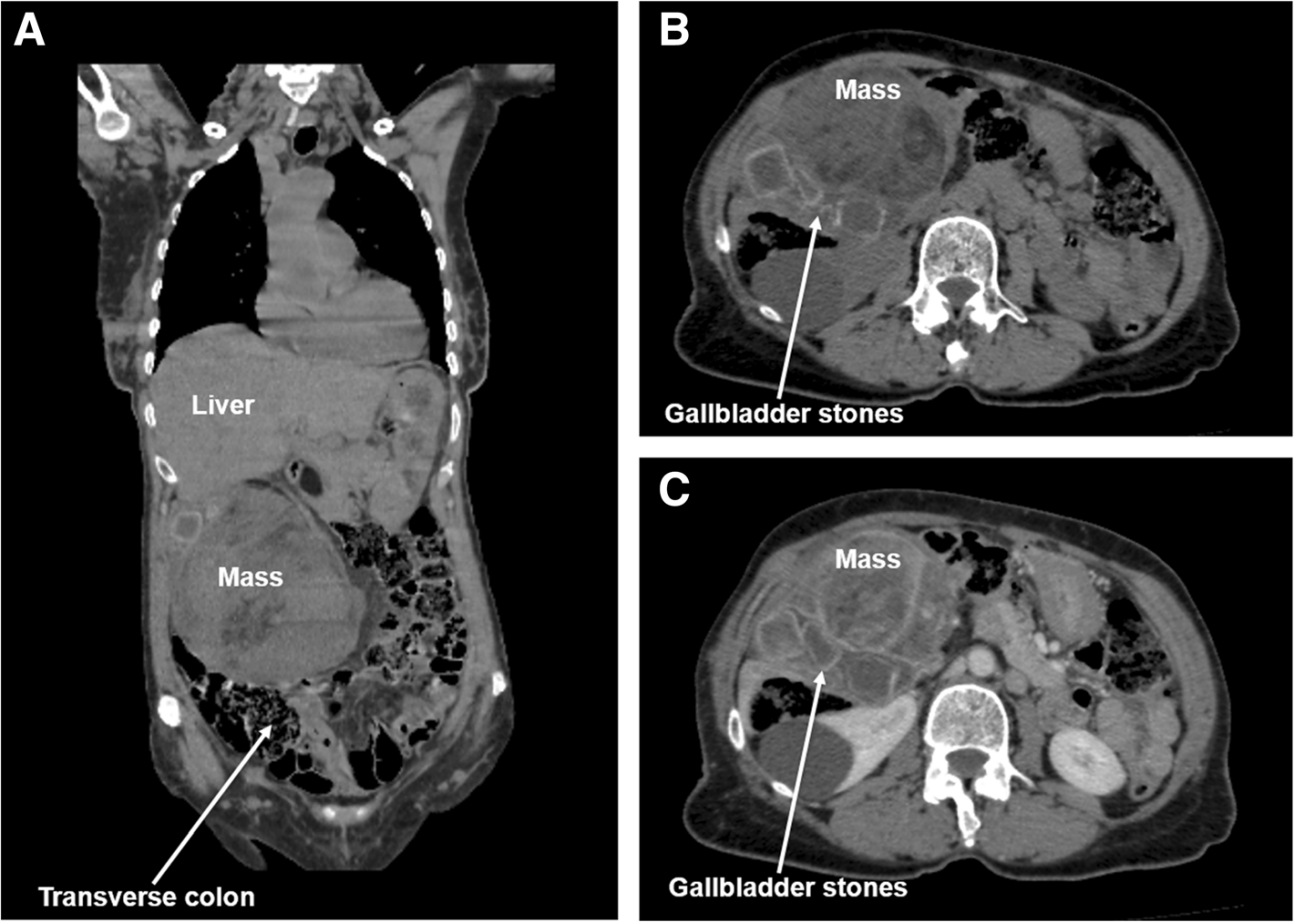
Preoperative CT scans: coronal section of non-enhanced CT image showing a large mass adhered to the liver, pushing the transverse colon down (**a**). Transverse section imaging showing three large gallbladder stones without (**b**) and with (**c**) CT contrast enhancement

A 71-year-old woman was referred to the Surgical Oncology Division at the Napoleão Laureano Hospital in João Pessoa, Brazil, presenting with a mass at the right side of the abdomen, associated with fever. She presented dyspeptic complaints and an isolated episode of acute pain in the upper abdomen, with radiation to the back, 20 days before the consultation. At admission, no signs of jaundice were found. An abdominal mass with elastic consistency was palpated at the right hypochondrium, 20 cm below the last rib. Laboratory tests indicated the following measures: hemoglobin 9.5 g/dL, carcinoembryonic antigen (CEA 1.96 ng/mL), serum C-reactive protein (123.8 mg/L), serum alkaline phosphatase (ALP 244 U/L), gamma-glutamyl transpeptidase (GGT 134 U/L), AST, ALT, albumin, and bilirubin within normal range. Abdominal ultrasonography (US) evidenced the presence of a solid, hypoechoic heterogeneous mass, measuring 14.2 × 9.5 × 13.8 cm, located on the right flank of the abdomen. Computed tomography (CT) showed extensive lobular formation (14.7 × 14.4 × 10.5 cm), exhibiting irregular enhancement and areas of fat density in the right side of the abdominal cavity, without cleavage plan with the gallbladder. The mass was adherent to the inferior border of the right hepatic lobe, bulging the ipsilateral abdominal wall and compressing the transverse colon, modifying its anatomic position (Fig. 1a). Three large gallbladder stones were identified (Fig. 1b, c). The CT report suggested gallbladder liposarcoma and cholelithiasis. Thoracic CT scan was normal. Based on these findings, on 3 February 2018, a laparotomy was indicated.

### Surgical findings and anatomopathological examination results

**Fig. 2:**
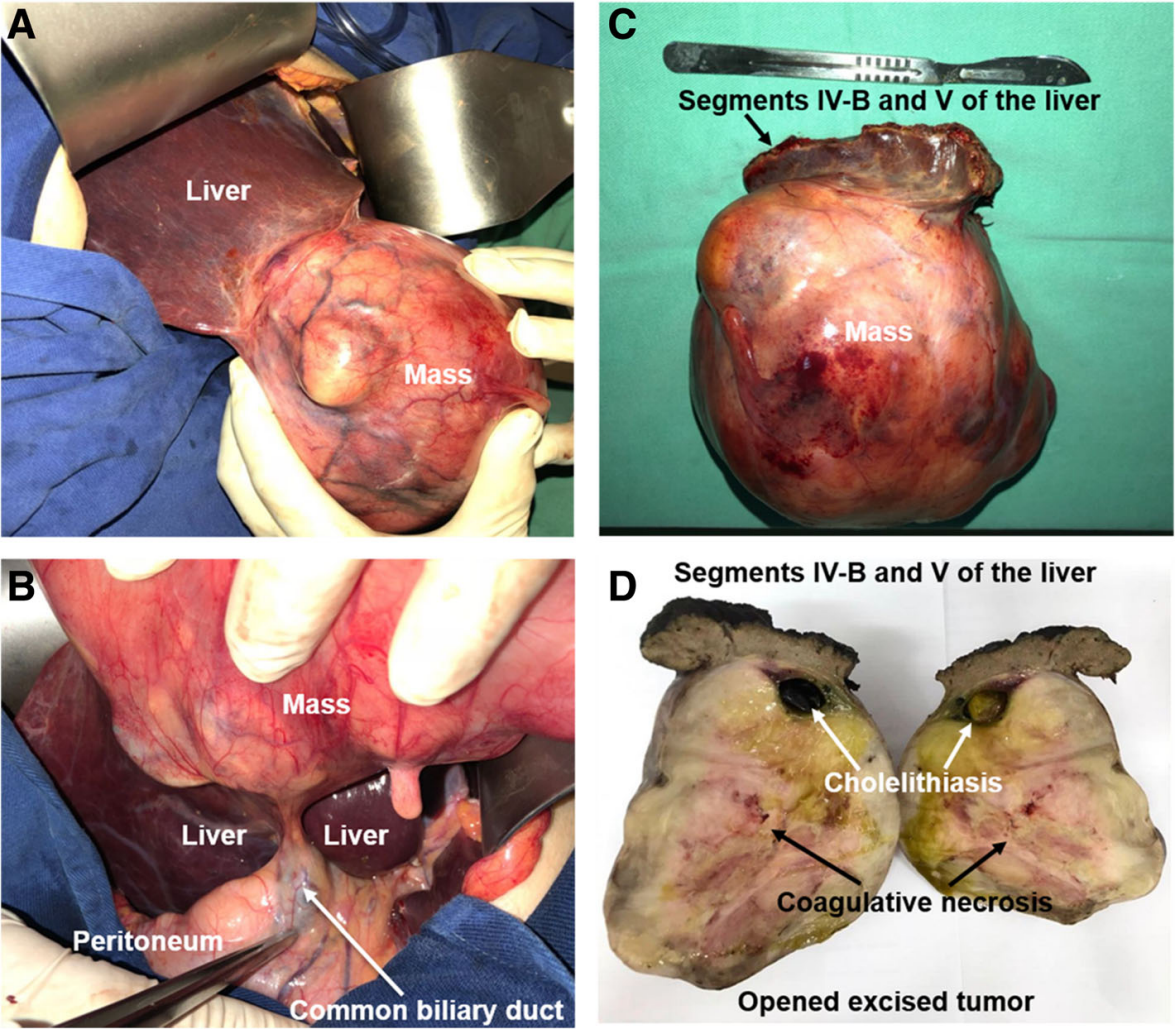
Gallbladder tumor adhered to the right hepatic lobe (**a**). Peritoneum and common biliary duct without tumor invasion (**b**). Gallbladder tumor resected with segments IV-B and V of the liver (**c**). Opened excised tumor with areas of coagulative necrosis and cholelithiasis (**d**)

An extensive gallbladder tumor associated with focal liver invasion was observed (Fig. 2a). The cystic and biliary ducts were free from neoplastic invasion, and there were no signs of peritoneal spread nor of other organ metastases (Fig. 2b). A cholecystectomy associated with resection of segments IV-B and V of the liver was done (Fig. 2c). Intraoperative frozen sections of the opened excised tumor (Fig. 2d) were compatible with gallbladder sarcoma. Therefore, transoperative lymphadenectomy was not performed.

**Fig. 3:**
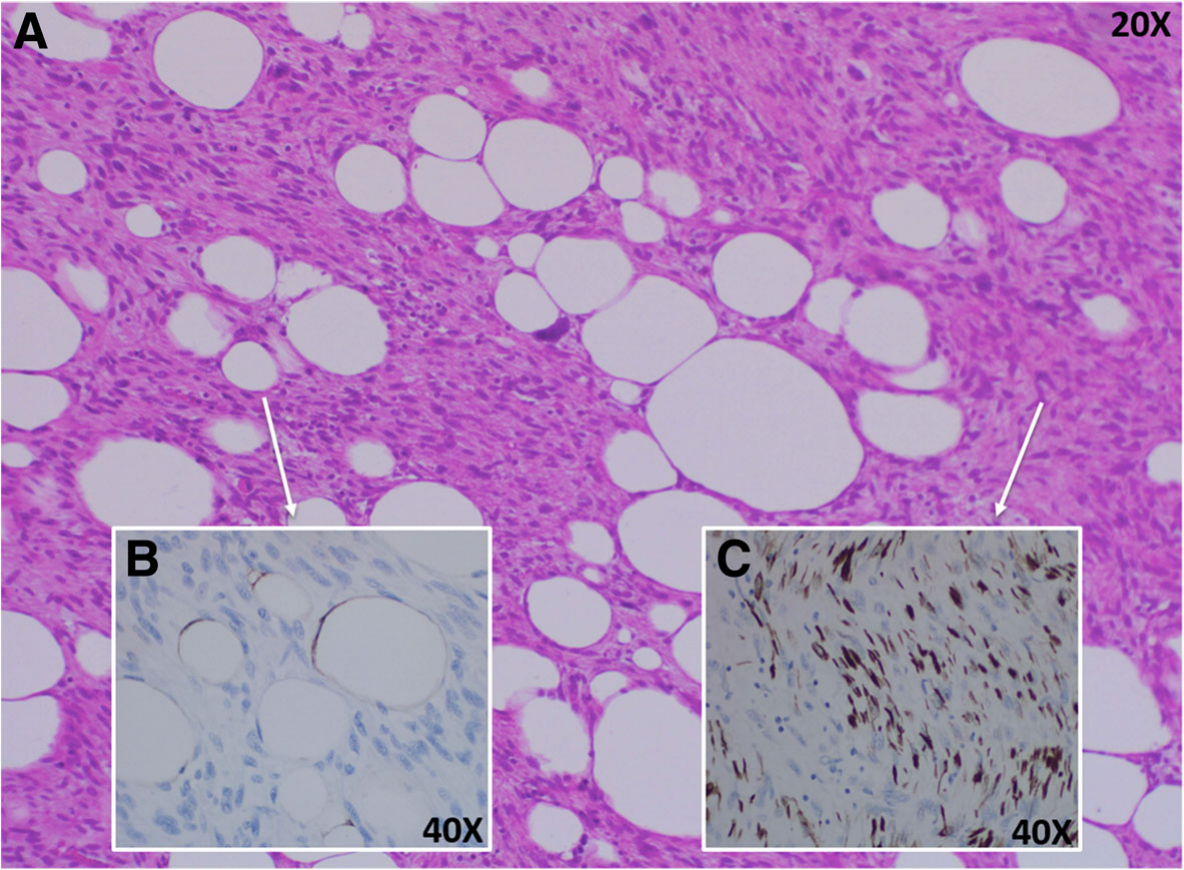
Dedifferentiated liposarcoma of the gallbladder stained with hematoxylin and eosin (H&E) (**a**). Foci of heterologous leiomyosarcomatous differentiation with immunohistochemical (IHC) staining for S100 Protein (**b**) and undifferentiated fusocellular areas of high histological grade with IHC staining for desmin (**c**)

The patient was released 7 days after the surgical procedure and the anatomopathological examination and immunohistochemistry confirmed dedifferentiated liposarcoma (Fig. 3a) with foci of heterologous leiomyosarcomatous differentiation (Fig. 3b) and undifferentiated fusocellular areas of high histological grade (Fig. 3c), stage T3N0M0. Other immunohistochemical staining were studied, such as Pan-cytokeratin (AE1/AE3), epithelial membrane antigen (EMA), and high molecular weight cytokeratin (34betaE12), in order to refute the possibility of a carcinosarcoma of the gallbladder. All of them were negative.

### Post-operative course

**Fig. 4:**
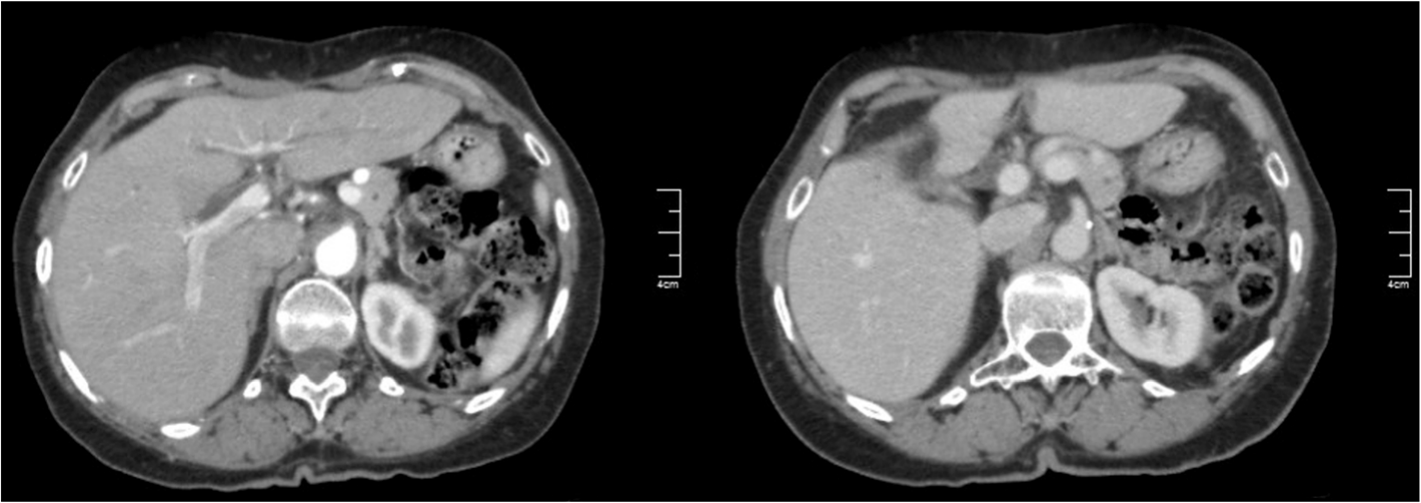
Postoperative CT scans: transverse section imaging showing no signs of recurrence 8 months after surgery

At 8 months after the procedure, the patient is free of disease, presenting no signs of recurrence nor metastasis (Fig. 4).

## Discussion and conclusion

Liposarcoma of the gallbladder is one of the least common sarcomas [1], with only five cases reported in the literature according to our knowledge (Table 1). The mean age at diagnosis of LPS among the five cases reported in the literature and ours is 67.17 ± 10.11 years, and there is a prevalence of 83.33% of female patients.

**Table 1:** Previous reported cases of Liposarcoma of the gallbladder

Author	Age	Sex	Type	Therapy	Prognosis
Our case	71	F	DL	Cholecystectomy + resection of segments IV-B and V of the liver	Alive without signs of recurrence 8 months after surgery
Ma et al. [3]	70	F	MLS	Cholecystectomy	Not described
Hamada et al. [9]	49	F	PL	Cholecystectomy	Recurrence and metastasis to the liver. Alive 3 years and 6 months after the first operation
Bader and Vallon [10]	79	M	MLS	Cholecystectomy	Peritoneal dissemination. Died 2 years after the first operation
Husain et al. [2]	64	F	MLS	^a^Chemotherapy	^b^Patient died of the disease
Husain et al. [2]	70	F	MLS	Not described	Not described

^a^Not specified if associated or not to a cholecystectomy

^b^Not specified how long after the diagnosis

The WHO classification for soft tissue neoplasms divides liposarcoma into four types: atypical lipomatous tumor (ALT), myxoid liposarcoma (MLS), pleomorphic liposarcoma (PL), and dedifferentiated liposarcoma (DL) [6]. This classification reflects the spectrum of presentations of these diseases related to clinical progression, therapeutic sensitivity, and morphologic features [5].

Regarding the localized disease, surgical resection with clear margins remains to be the mainstay of treatment for LPS [5, 7]. The use of systemic therapy does not improve the course of the disease, except in cases of MLS, which is more chemosensitive and radiosensitive than the others subtypes [5, 8]. Similarly to the PL, the DL is a high grade and aggressive tumor, typically chemoinsensitive and radioinsensitive, in which cases the benefit of systemic therapy has been reported to be minimal [5, 7].

Livingstone et al. assessed the response of retroperitoneum DL to chemotherapy by the traditional RECIST (Response Evaluation Criteria in Solid Tumors) criteria in addition to an exploratory analysis of vascular alterations within the tumor. Using this methodology to assess response, they found that, in some selected cases, such as recurrent disease and unresectable tumors, DL can benefit from a combination chemotherapy [7].

The prognosis of patients with gallbladder liposarcoma is poor, because these are tumors with an aggressive behavior and, generally, present high rates of recurrence and metastasis [5]. Taking this scenario into consideration, it is expected that, in the future, the patient of our case may require further surgical procedures in order to resect possible sites of metastasis, such as happened in the cases described by Hamada et al. [9] and Bader et al. [10].

The main limitation of our case is the fact that we do not have a longer term follow-up of this patient. Therefore, we could not determine the prognosis of the case.

To sum up, among the five cases of gallbladder liposarcoma ever reported in the literature to date, only two histological types were identified (one PL [9] and four MLS [3, 10]). Our case stands as the first case of subtype DL of the gallbladder to be reported.

## Abbreviations

ALT: Atypical lipomatous tumor
DL: Dedifferentiated liposarcoma
MLS: Myxoid liposarcoma
PL: Pleomorphic liposarcoma

## References

[1] Zimmermann A (2016). Mesenchymal tumors of the gallbladder. Tumors and tumor-like lesions of the hepatobiliary tract.

[2] Husain EA, Prescott RJ, Haider SA (2009). Gallbladder sarcoma: a clinicopathological study of seven cases from the UK and Austria with emphasis on morphological subtypes. Dig Dis Sci.

[3] Ma Y, Wei S, Peker D (2014). An extremely rare primary gallbladder myxoid liposarcoma associated with amplification of DDIT3 gene. J Gastrointestin Liver Dis.

[4] Enzinger FM, Weiss SW (1988). Soft tissue tumors.

[5] Lee ATJ, Thway K, Huang PH, Jones RL (2018). Clinical and molecular spectrum of liposarcoma. J Clin Oncol.

[6] Fletcher CD, Bridge JA, Hogendoorn P, Mertens F, World Health Organization, International Agency for Research on Cancer (2013). WHO classification of tumours of soft tissue and bone.

[7] Livingstone JA, Bugano DB, Lin H (2017). Role of chemotherapy in dedifferentiated liposarcoma of the retroperitoneum: defining the benefit and challenges of the standard. Sci Rep.

[8] Gronchi A, Bui BN, Donvalot S (2012). Phase II clinical trial of neoadjuvant trabectedin in patients with advanced localized myxoid liposarcoma. Ann Oncol.

[9] Hamada T, Yamagiwa K, Okanami Y (2006). Primary liposarcoma of gallbladder diagnosed by preoperative imagings: a case report and review of literature. World J Gastroenterol.

[10] Bader H, Vallon H (1983). Liposarcoma of the gallbladder and the peritoneum. A case report. Zentralbl Allg Pathol.

